# Complete genome sequence of *Brachybacterium faecium* type strain (Schefferle 6-10^T^)

**DOI:** 10.4056/sigs.492

**Published:** 2009-07-20

**Authors:** Alla Lapidus, Rüdiger Pukall, Kurt LaButtii, Alex Copeland, Tijana Glavina Del Rio, Matt Nolan, Feng Chen, Susan Lucas, Hope Tice, Jan-Fang Cheng, David Bruce, Lynne Goodwin, Sam Pitluck, Manfred Rohde, Markus Göker, Amrita Pati, Natalia Ivanova, Konstantinos Mavrommatis, Amy Chen, Krishna Palaniappan, Patrik D'haeseleer, Patrick Chain, Jim Bristow, Jonathan A. Eisen, Victor Markowitz, Philip Hugenholtz, Nikos C. Kyrpides, Hans-Peter Klenk

**Affiliations:** 1 DOE Joint Genome Institute, Walnut Creek, California, USA; 2 DSMZ - German Collection of Microorganisms and Cell Cultures GmbH, Braunschweig, Germany; 3 Los Alamos National Laboratory, Bioscience Division, Los Alamos, New Mexico, USA; 4 HZI - Helmholtz Centre for Infection Research, Braunschweig, Germany; 5 Biological Data Management and Technology Center, Lawrence Berkeley National Laboratory, Berkeley, California, USA; 6 Lawrence Livermore National Laboratory, Livermore, California, USA; 7 University of California Davis Genome Center, Davis, California, USA

**Keywords:** mesophile, free-living, non-pathogenic, aerobic, rod-coccus growth cycle, uric acid degradation, *Dermabacteraceae*

## Abstract

*Brachybacterium faecium* Collins *et al*. 1988 is the type species of the genus, and is of phylogenetic interest because of its location in the *Dermabacteraceae,* a rather isolated family within the actinobacterial suborder *Micrococcineae*.* B. faecium* is known for its rod-coccus growth cycle and the ability to degrade uric acid. It grows aerobically or weakly anaerobically. The strain described in this report is a free-living, nonmotile, Gram-positive bacterium, originally isolated from poultry deep litter. Here we describe the features of this organism, together with the complete genome sequence, and annotation. This is the first complete genome sequence of a member of the actinobacterial family *Dermabacteraceae*, and the 3,614,992 bp long single replicon genome with its 3129 protein-coding and 69 RNA genes is part of the *** G****enomic* *** E****ncyclopedia of* *** B****acteria and* *** A****rchaea * project.

## Introduction

**Figure 1:**
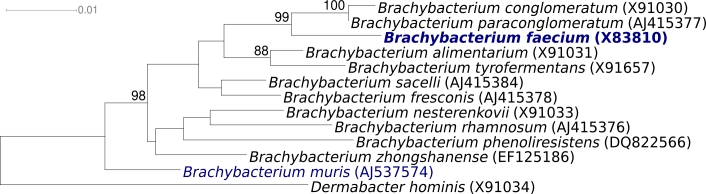
Phylogenetic tree of *B. faecium* Schefferle 6-10^T^ and all type strains of the genus *Brachybacterium*, inferred from 1408 aligned characters [2] of the 16S rRNA sequence under the maximum likelihood criterion [3,4]. The tree was rooted with *Dermabacter hominis*, another member of the family *Dermabacteraceae*. The branches are scaled in terms of the expected number of substitutions per site. Numbers above branches are support values from 1000 bootstrap replicates, if larger than 60%. Strains with a genome-sequencing project registered in GOLD [5] are printed in blue; published genomes in bold.

Strain Schefferle 6-10^T^ (DSM 4810 = ATCC 43885 = JCM 11609 = NCIMB 9860) is the type strain of *Brachybacterium faecium*, which is the type species of the genus *Brachybacterium* [1] (Figure 1). *B. faecium* was described by Collins *et al*. in 1988 [1] as Gram-positive and nonmotile. The organism is of significant interest for its position in the tree of life where the rapidly growing genus *Brachybacterium* (12 species) is located in the actinobacterial family *Dermabacteraceae* [6].

Only two accompanying strains (Schefferle 3-8 = NCIMB 9859, and Schefferle 7-11 = NCIMB 9861) were initially reported from the poultry deep litter from which the type strain Schefferle 6-10^T^ was isolated [7]. Both strains were later reclassified as members of other *Brachybacterium* species: *B. conglomeratum* (NCIMB 9859) and *B. paraconglomeratum* (NCIMB 9861). Some closely related isolates with more than 98.5% 16S rRNA gene sequence identity were reported from marine sediments (JH107; FJ572028), deep-sea sediments (PB10; DQ643203), and living room air (Gauze_W_12_19; FJ267545). Only four sequences from uncultured bacteria are accessible via EMBL, showing at least 98% sequence identity to *B. faecium*. These sequences were derived from the analyses of urban aerosols (DQ129569), human vaginal epithelium (AY959187), floor dust (FM872846), and water 20m downstream of manure (AY212613). No phylotypes from environmental screening or genomic surveys could be linked to *B. faecium* (as of February 2009). Here we present a summary classification and a set of features for *B. faecium* Schefferle 6-10^T^ (Table 1), together with the description of the complete genomic sequencing and annotation.

**Table 1:** Classification and general features of *B. faecium* Schefferle 6-10^T^ based on MIGS recommendations [8]

MIGS ID	Property	Term	Evidence code
	Current classification	Domain *Bacteria* Phylum *Actinobacteria* Class *Actinobacteria* Order *Actinomycetales* Family *Dermabacteraceae* Genus *Brachybacterium* Species *Brachybacterium faecium* Type strain Schefferle 6-10	TAS [6] TAS [6] TAS [6] TAS [1] TAS [1]
	Gram stain	positive	TAS [1]
	Cell shape	varies; rod-coccus growth cycle	TAS [1]
	Motility	nonmotile	TAS [1]
	Sporulation	non-sporulating	TAS [1]
	Temperature range	mesophilic	TAS [1]
	Optimum temperature	25-30°C	TAS [1]
	Salinity	5g NaCl/L	TAS [1]
MIGS-22	Oxygen requirement	aerobic; very weak growth under anaerobic conditions	TAS [1]
	Carbon source	glucose, maltose, mannose, cellobiose	TAS [1]
	Energy source	starch	NAS
MIGS-6	Habitat	deep litter (soil)	TAS [7]
MIGS-15	Biotic relationship	free-living	NAS
MIGS-14	Pathogenicity	none	NAS
	Biosafety level	1	TAS [9]
	Isolation	poultry deep litter	TAS [7]
MIGS-4	Geographic location		TAS [7]
MIGS-5	Sample collection time	about 1966	TAS [7]
MIGS-4.1 MIGS-4.2	Latitude – Longitude	not reported	
MIGS-4.3	Depth	not reported	
MIGS-4.4	Altitude	not reported	

Evidence codes - IDA: Inferred from Direct Assay (first time in publication); TAS: Traceable Author Statement (i.e., a direct report exists in the literature); NAS: Non-traceable Author Statement (i.e., not directly observed for the living, isolated sample, but based on a generally accepted property for the species, or anecdotal evidence). These evidence codes are from the Gene Ontology project [10]. If the evidence code is IDA, then the property should have been directly observed, for the purpose of this specific publication, for a live isolate by one of the authors, or an expert or reputable institution mentioned in the acknowledgements.

## Classification and features

**Figure 2:**
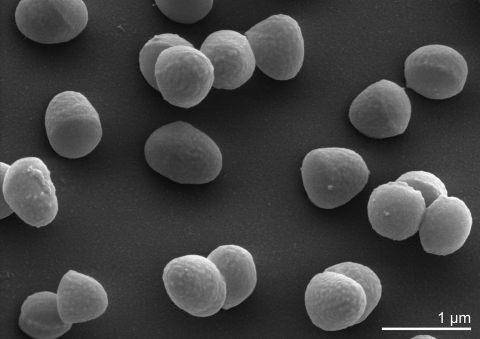
Scanning electron micrograph of *B. faecium* Schefferle 6-10^T^

*B. faecium* Schefferle 6-10^T^ cells vary in shape and exhibit a rod-coccus growth cycle, which is not atypical of this evolutionary group. Cells in the stationary phase are predominantly coccoid (Figure 2), whereas cells in fresh cultures are irregular, slender rods [1]. Cells are frequently arranged at an angle to give V-formations [1] (Figure 2). *B. faecium* cells are non-acid fast and do not form endospores [1]. *B. faecium* is essentially aerobic, but is also capable of very weak growth under anaerobic conditions [1].

*B. faecium* is capable of degrading uric acid, and fermenting cellobiose, glucose, maltose, and mannose, but not cellulose, chitin, or gelatin. The optimal growth temperature is 25-30°C. Nitrate is reduced to nitrite by some *B. faecium* strains [1] as a candidate for terminal electron acceptor during anaerobic growth.

Figure 1 shows the phylogenetic neighborhood of *B. faecium* strain Schefferle 6-10^T^ in a 16S rRNA based tree. The sequences of the three 16S rRNA genes in the *B. faecium* Schefferle 6-10^T^ genome differ by up to two nucleotides (nts) from each other, and by three nts from the reference sequence of strain DSM 4810 (X91032). The slight differences between the genome data and the previously reported 16S rRNA gene sequence is most likely due to sequencing errors in the previously reported sequence data.

### Chemotaxonomy

Strain Schefferle 6-10^T^ was originally described as a coryneform bacterium. This descriptive term applies to a diverse range of taxa and indicates that the comparisons made in the original publication need to be reviewed. The murein of *B. faecium* contains *meso*-diaminopimelic acid, alanine and glutamic acid. The strain possesses a type A4γ peptidoglycan, type A31.2 according to the German Collection of Microorganisms and Cell Cultures. Galactose and glucose are the cell wall sugars [1]. As in other *Brachybacterium* strains, the fatty acid pattern of strain Schefferle 6-10^T^ is dominated by branched-chain saturated anteiso- (ai-) fatty acids: ai-C_15:0_ (40%), ai-C_17:0_ (37%), and C_16:0_ and iso-C_16:0_ 7.5%, each, with smaller amounts of iso-C_15:0_ (3.5%), iso-C_17:0_ (2.0%) [1]. Straight chain and unsaturated fatty acids are absent [1]. As usual for most members of the *Actinomycetales*, mycolic acids were not reported [1]. A menaqui-  none with seven isoprene units (MK-7) predominates (88%) complemented by 11% MK-8 [1]. Phosphatidylglycerol and diphosphatidylglycerol were identified as the dominant polar lipids, together with several glycolipids and an unknown phospholipid [1]. The R_f_ values of the glycolipids suggest that they contain different numbers of sugars (one, two or possibly three) and may also show differences in the nature and linkage of the sugars. It is not known whether these glycolipids are based on a diglyceride or whether they contain an acylated sugar, directly linked to a monoglyceride. The chemical composition is typical of members of the genus *Brachybacterium* and similar, but not identical with the members of the only other genus placed in the family *Dermabacteraceae*, *Dermabacter*. In addition to cytochrome aa3, *B. faecium* possesses cytochrome d_626_, and cytochrome c_550_ [11].

## Genome sequencing and annotation

### Genome project history

This organism was selected for sequencing on the basis of its phylogenetic position, and is part of the *** G****enomic* *** E****ncyclopedia of* *** B****acteria and* *** A****rchaea * project. The genome project is deposited in the Genomes OnLine Database [5] and the complete genome sequence in GenBank (CP001643). Sequencing, finishing and annotation were performed by the DOE Joint Genome Institute (JGI). A summary of the project information is shown in Table 2.

**Table 2:** Genome sequencing project information

MIGS ID	Property	Term
MIGS-31	Finishing quality	Finished
MIGS-28	Libraries used	Two genomic libraries: 8kb pMCL200 and fosmid pcc1Fos Sanger libraries One 454 pyrosequence standard library and one Illumina library
MIGS-29	Sequencing platforms	ABI3730, 454 GS FLX, Illumina GA
MIGS-31.2	Sequencing coverage	10x Sanger; 40x pyrosequence
MIGS-30	Assemblers	Newbler version 1.1.02.15, PGA
MIGS-32	Gene calling method	Genemark 4.6b, tRNAScan-SE-1.23, infernal 0.81
	Genbank ID	CP001643
	Genbank Date of Release	N/A
	GOLD ID	Gi02066
	NCBI project ID	17026
	Database: IMG-GEBA	2500868055
MIGS-13	Source Material Identifier	DSM 4810
	Project relevance	Tree of Life, GEBA

### Growth conditions and DNA isolation

*B. faecium* Schefferle 6-10^T^, DSM 4810, was grown in DSMZ medium 92 (with 3% trypticase soy broth, 0.3% yeast extract) at 28°C. DNA was isolated from 1-1.5 g of cell paste using Qiagen Genomic 500 DNA Kit (Qiagen, Hilden, Germany) without modification of the manufacturer’s protocol for cell lysis.

### Genome sequencing and assembly

The genome was sequenced using a combination of Sanger, 454 and Illumina sequencing platforms. All general aspects of library construction and sequencing performed at the JGI can be found on the JGI website. 454 Pyrosequencing reads were assembled using the Newbler assembler version 1.1.02.15 (Roche). Large Newbler contigs were broken into 4,074 overlapping fragments of 1,000 bp and entered into the assembly as pseudo-reads. The sequences were assigned quality scores based on Newbler consensus q-scores with modifications to account for overlap redundancy and to adjust inflated q-scores. A hybrid 454/Sanger assembly was made using the PGA assembler. Possible mis-assemblies were corrected and gaps between contigs were closed by custom primer walks from sub-clones or PCR products. 258 Sanger finishing reads were produced. Illumina reads were used to improve the final consensus quality using an in-house developed tool (the Polisher). The error rate of the completed genome sequence is less than 1 in 100,000. Together all sequence types provided 50x coverage of the genome.

### Genome annotation

Genes were identified using GeneMark [12] as part of the genome annotation pipeline in the Integrated Microbial Genomes Expert Review (IMG-ER) system [13], followed by a round of manual curation using the JGI GenePRIMP pipeline. The predicted CDSs were translated and used to search the National Center for Biotechnology Information (NCBI) nonredundant database, UniProt, TIGRFam, Pfam, PRIAM, KEGG, COG, and InterPro databases. The tRNAScanSE tool [15] was used to find tRNA genes, whereas ribosomal RNAs were found by using the tool RNAmmer [16]. Other non-coding RNAs were identified by searching the genome for the Rfam profiles using INFERNAL (v0.81) [17]. Additional gene prediction analysis and manual functional annotation was performed within the Integrated Microbial Genomes (IMG) platform [18].

### Metabolic network analysis

The metabolic Pathway/Genome Database (PGDB) was computationally generated using Pathway Tools software version 12.5 [19] and MetaCyc version 12.5 [20], based on annotated EC numbers and a customized enzyme name mapping file. It has undergone no subsequent manual curation and may contain errors, similar to a Tier 3 BioCyc PGDB [21].

### Genome properties

**Figure 3:**
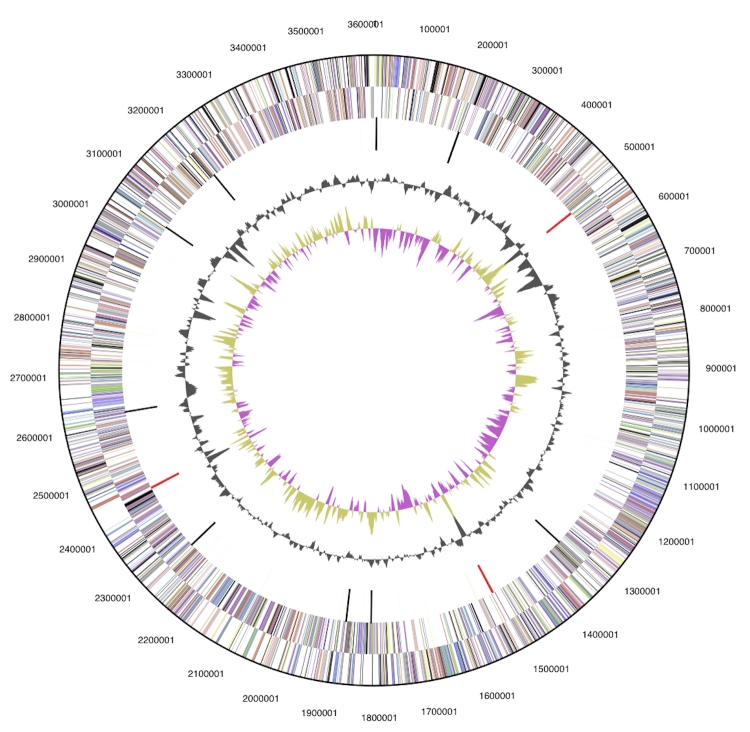
Graphical circular map of the genome. From outside to the center: Genes on forward strand (color by COG categories), Genes on reverse strand (color by COG categories), RNA genes (tRNAs green, rRNAs red, other RNAs black), GC content, GC skew.

**Figure 4:**
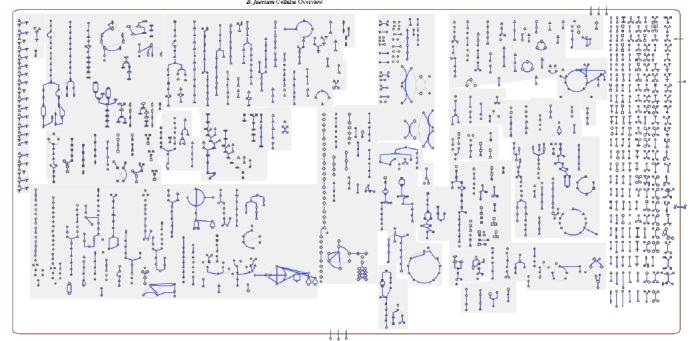
Schematic cellular overview of all pathways of the *B. faecium* strain Schefferle 6-10^T^ metabolism. Nodes represent metabolites, with shape indicating class of metabolite. Lines represent reactions.

The genome is 3,614,992 bp long and comprises one circular chromosome with a 72.1% GC content (Table 3 and Figure 3). Of the 3,198 genes predicted, 3,129 were protein coding genes, and 69 RNAs. Sixty pseudogenes were also identified. The majority of genes (77.3%) of the genes were assigned with a putative function while the remaining ones are annotated as hypothetical proteins. The properties and the statistics of the genome are summarized in Table 3. The distribution of genes into COGs functional categories is presented in Table 4, and a cellular overview diagram is presented in Figure 4, followed by a summary of metabolic network statistics shown in Table 5.

**Table 3:** Genome Statistics

Attribute	Value	% of Total
Genome size (bp)	3,614,992	
DNA Coding region (bp)	3,287,735	90.95%
DNA G+C content (bp)	2,604,449	72.05%
Number of replicons	1	
Extrachromosomal elements	0	
Total genes	3198	
RNA genes	69	2.16%
rRNA operons	3	
Protein-coding genes	3129	97.84%
Pseudo genes	60	1.88%
Genes with function prediction	2473	77.33%
Genes in paralog clusters	347	10.85%
Genes assigned to COGs	2371	74.14%
Genes assigned Pfam domains	2440	76.30%
Genes with signal peptides	697	21.79%
Genes with transmembrane helices	836	26.14%
CRISPR repeats	0	

**Table 4:** Number of genes associated with the 21 general COG functional categories

Code	Value	%	Description
J	163	5.2	Translation, ribosomal structure and biogenesis
A	1	0.0	RNA processing and modification
K	204	6.5	Transcription
L	125	4.0	Replication, recombination and repair
B	1	0.0	Chromatin structure and dynamics
D	19	0.6	Cell cycle control, mitosis and meiosis
Y	0	0.0	Nuclear structure
V	53	1.7	Defense mechanisms
T	88	2.8	Signal transduction mechanisms
M	127	4.1	Cell wall/membrane biogenesis
N	2	0.1	Cell motility
Z	1	0.0	Cytoskeleton
W	0	0.0	Extracellular structures
U	25	0.8	Intracellular trafficking and secretion
O	68	2.2	Posttranslational modification, protein turnover, chaperones
C	136	4.3	Energy production and conversion
G	366	11.7	Carbohydrate transport and metabolism
E	261	8.3	Amino acid transport and metabolism
F	84	2.7	Nucleotide transport and metabolism
H	107	3.4	Coenzyme transport and metabolism
I	92	2.9	Lipid transport and metabolism
P	145	4.6	Inorganic ion transport and metabolism
Q	44	1.4	Secondary metabolites biosynthesis, transport and catabolism
R	333	10.6	General function prediction only
S	189	6.0	Function unknown
-	758	24.2	Not in COGs

**Table 5:** Metabolic Network Statistics

**Attribute**	Value
Total genes	3198
Enzymes	674
Enzymatic reactions	1031
Metabolic pathways	218
Metabolites	758
